# Supplementary material for: Unimolecularly thick monosheets of vinyl polymers fabricated in metal–organic frameworks
Source: Nat Commun. 2020 Jul 17;11:3573. doi: 10.1038/s41467-020-17392-1 (PMC7367882; doi:10.1038/s41467-020-17392-1)
Supplement: Supplementary file 1 — Supplementary Information [file 41467_2020_17392_MOESM1_ESM.pdf]

Supplementary Information

Unimolecularly thick monosheets of vinyl polymers fabricated in metal–organic frameworks

Hosono *et al*.

1. Supplementary Figures

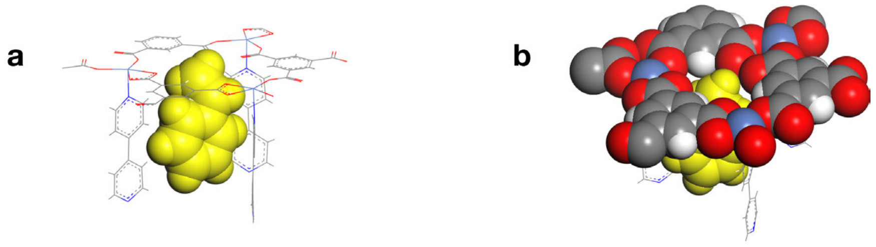

Supplementary Figure 1. (a) Graphical depiction of St placed in the nanochannel of **1** (**1**⊃St). The St molecule cannot pass through the very narrow aperture of $[Ni(Hbtc)]_n$ layer. Thus, inter-layer diffusion of St molecules is not allowed in **1**⊃St. The same situation applies to the system of **1**⊃MMA (Supplementary Figure 7). (b) Another depiction of panel (a) using space-filling models.

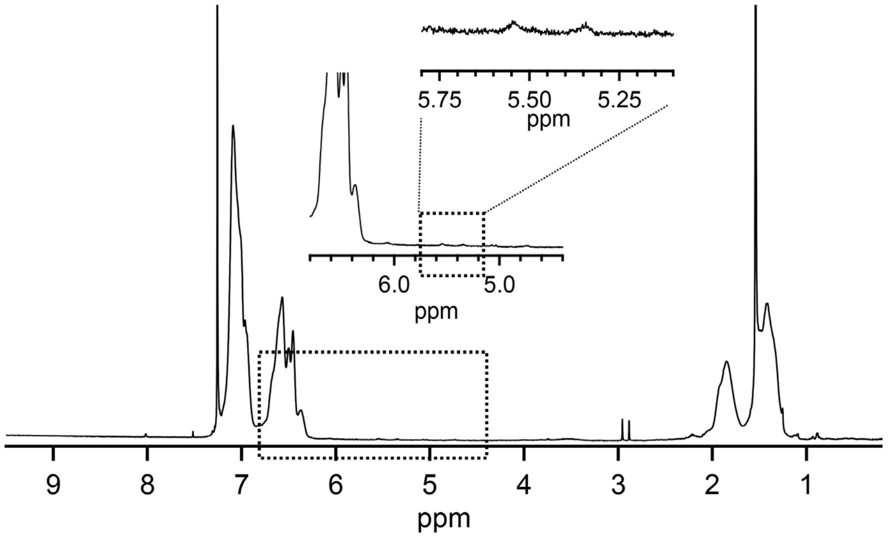

Supplementary Figure 2. ^{1}H NMR spectrum ($CDCl_3$) of isolated PSt-**1** measured at 25 °C. No proton signal originating from the residual ligands (Hbtc and bpy) was observed, which supports the successful removal of MOF host. In addition, small signals of end-groups derived from AIBN were observed in 1.1–0.9 ppm. Based on area integration analysis using the end groups and main chain peaks, an averaged degree of polymerization (*DP*) is calculated to be ~800 (M_n = ~83,000) when assuming linear chains without crosslinking. Here we made the assumption that all polystyrene chains are end-capped with the AIBN fragment at one end. Considering the absolute molecular weight of PSt-**1** (M_w = 298,000), this calculation provides us a useful aspect of molecular structure of PSt-**1** in which 3.6 linear chains with an average molecular weight of ~83,000 are crosslinked each other. A schematic illustration of plausible network structure is given in Supplementary Figure 6.

Insets show the magnified spectra of the proton signals in 5-6 ppm. Small peaks at 5.34 and 5.54 ppm can be attributed to the protons of unreacted methacrylate unit of EDMA (2H, integration area: A). The methylene protons of EDMA are observed at 3.5 ppm as a broad peak (4H, integration area: B). The ratio of those integration values (2A/B) becomes ~0.15, which indicates that the crosslinking degree is 0.85% = ~1%.

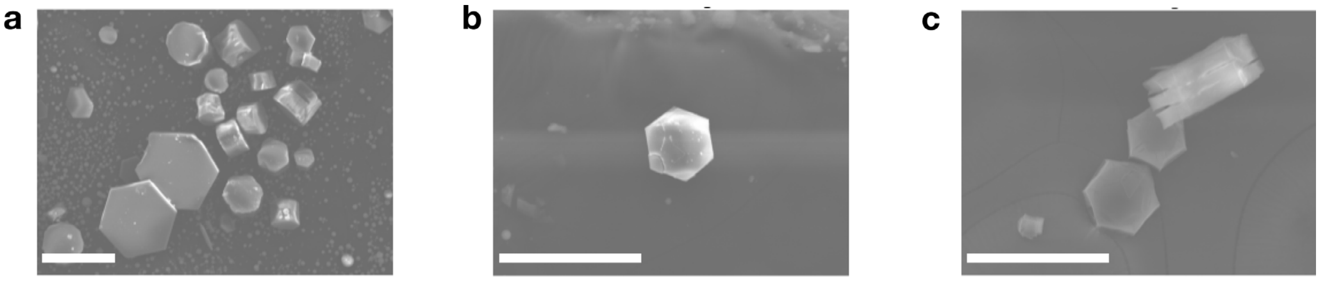

Supplementary Figure 3. SEM images of (a) **1**, (b) **1**⊃PSt, and (c) PSt-**1** immediately after isolation from the host MOF. Scale bars: 100 μm.

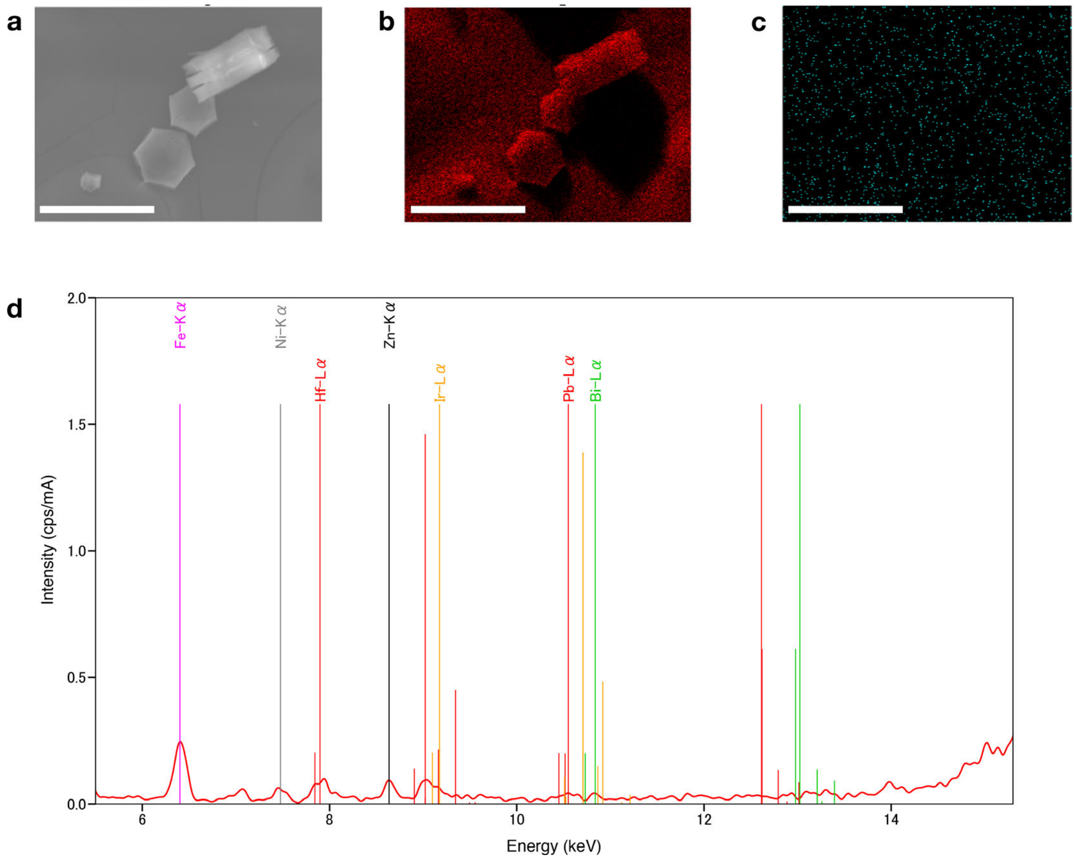

Supplementary Figure 4. (a) A SEM image of PSt-**1** particles and (b,c) SEM-EDX images of PSt-**1** particles with regard to (b) carbon and (c) nickel species. Scale bars: 100 μm. (d) XRF spectrum of PSt-**1**. The signal from Ni was negligible, supporting the complete removal of the MOF host. A Fe signal might be caused by stain-less steel which is used for the sample socket.

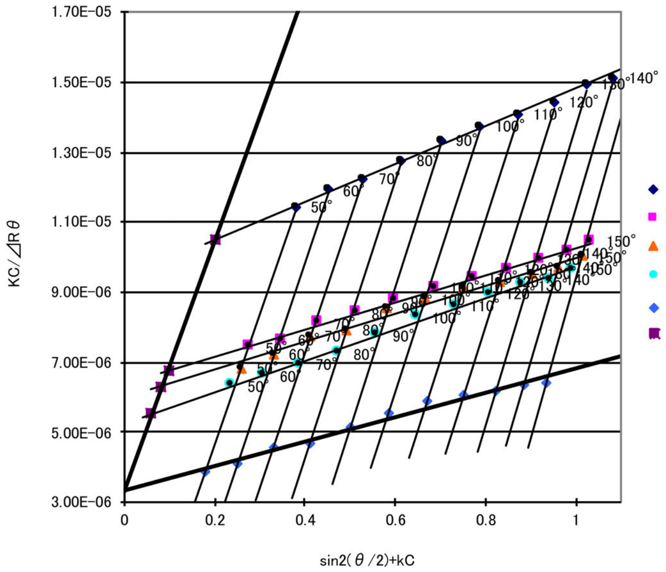

Supplementary Figure 5. Zimm-plot of PSt-**1** measured in toluene at given concentrations at 25 °C. M_w = 298,000 g mol^{-1}; R_g = 59.3 nm; $A_2 = 3.56 \times 10^{-3}$ mol·cm^3 g^{-2}.

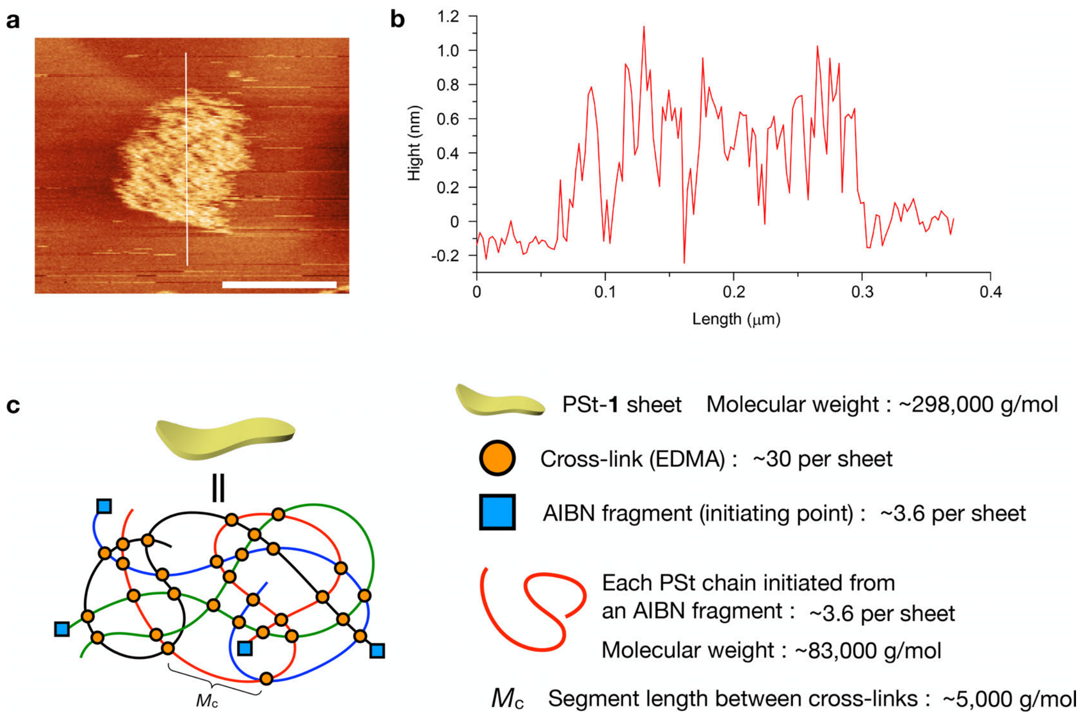

Supplementary Figure 6. (a) An AFM topographic image of PSt-**1** deposited on HOPG (Scale bar: 200 nm) and (b) the height profile along the white line in panel a. The cantilever, OMCL-AC200TSA (Olympus), with spring constant of 9 pN nm^{-1} and 150 kHz resonant frequency was used, which gives a milder condition than that is used for the AFM imaging of Figure 4. (c) A schematic illustration of speculated network structure of each PSt-**1** monosheet. The numbers of crosslinks and molecular weight values are calculated based on the experimental data determined by NMR and SLS analysis and a hypothesis that each chain has one AIBN end group.

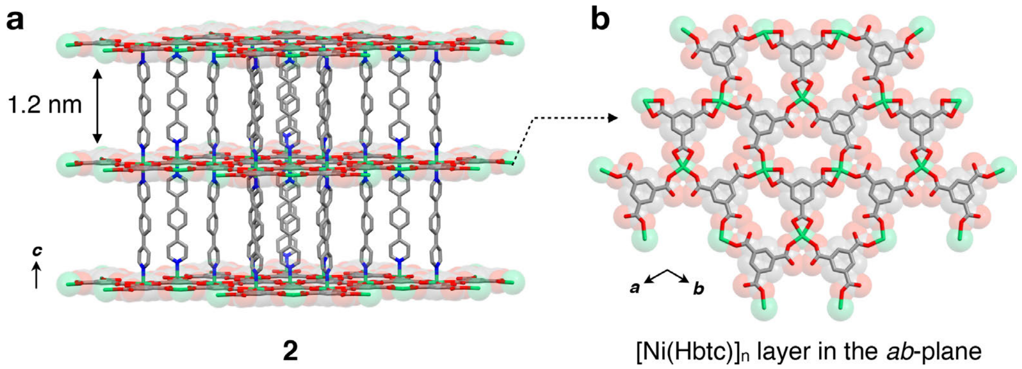

Supplementary Figure 7. (a) Crystal structure of **2**. (b) The structure of the $[Ni(Hbtc)]_n$ layer. Atoms: Ni (green), O (red), C (grey), and N (blue). H atoms are omitted for clarity.

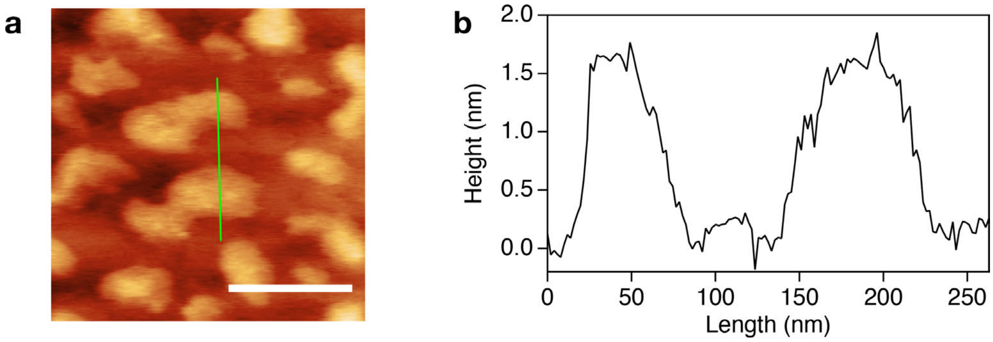

Supplementary Figure 8. (a) AFM image of PSt-**2** deposited on a HOPG substrate (Scale bar: 200 nm) and (b) height profile along the green line in panel (a). The sample was prepared by spin-cast on the substrate from a 0.1 μg mL^{-1} chloroform solution of PSt-**2**.

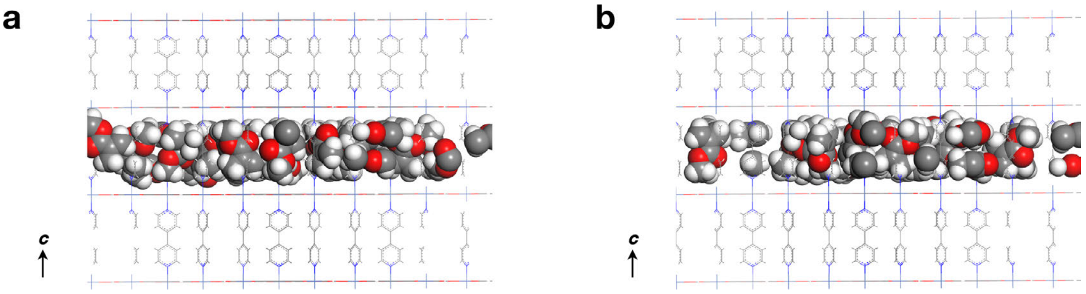

Supplementary Figure 9. MD simulations for **1**⊃MMA. (a) The initial structure of **1**⊃MMA composite. (b) A snapshot of the final structure of **1**⊃MMA composite after quenching the dynamics from 493 K to 293 K for 1000 ps under NVT condition. The MMA molecules do not leap across $[Ni(Hbtc)]_n$ layers into neighboring channels.

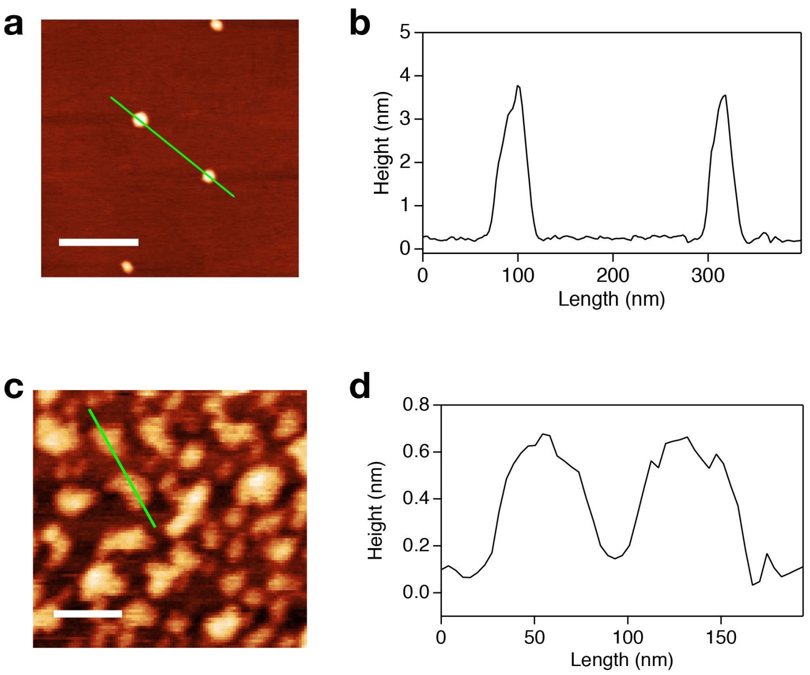

Supplementary Figure 10. (a) AFM image and (b) height profile of PMMA-**1** deposited on a HOPG substrate. Scale bar: 200 nm. (c) AFM image and (d) height profile of PMMA-**1** deposited on a mica substrate. Scale bar: 100 nm. Each height profile follows the green line in the corresponding AFM image. All materials were spin-cast on the given substrate from a 0.1 μg mL^{-1} chloroform solution.

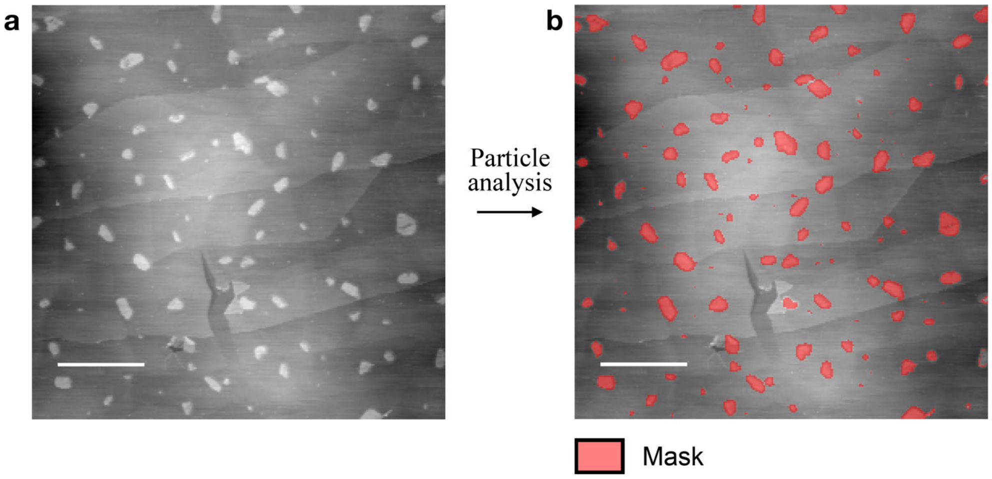

$$A \times H \times D_{\mathrm{PSt}} \times N_{\mathrm{A}} = \mathrm{MW}$$

A : Masked area
H : Sheet height (0.7 nm)
D_{PSt} : Density of polystyrene (1.04 g cm^{-3})
N_{A} : Avogadro's number
MW : Molecular weight of a sheet

Calculated M_{n} from AFM image
($N = 147$)
5.3×10^{6} g mol^{-1}

Supplementary Figure 11. The molecular weight of PSt-**1** was calculated by means of particle analysis on AFM data. (a) An AFM topographic image of dispersed monosheets of PSt-**1** deposited on HOPG surface. PSt-**1** monosheets with a wide variety of sizes (ca. 50-300 nm) are observed. Scale bar: 1 μm. (b) Particle analysis is applied to the AFM image of panel a. Scale bar: 1 μm. The area of

each PSt-**1** sheet is measured for N = 127 sheets and statistically analyzed to calculate the averaged molecular weight based on the given equation in the figure. The density of polystyrene of 1.04 g cm^{-3} was used. Although the AFM-based value is larger than the SLS-based value (298,000 g mol^{-1}), this can be explained by the difference in the way on analyses and lower density of polystyrene. Because of the imaging resolution limit, AFM is not able to visualize low-molecular weight fraction of PSt-**1**, which results in the overestimated M_n. In addition, the monosheets should have lower density compared with bulk polystyrene because of loose molecular packing due to the unimolecular thickness[5] and potential porosity in the mesh structure (Supplementary Figure 6 and 12). Therefore, both AFM- and SLS-based values could be in the range of the molecular weight distribution.

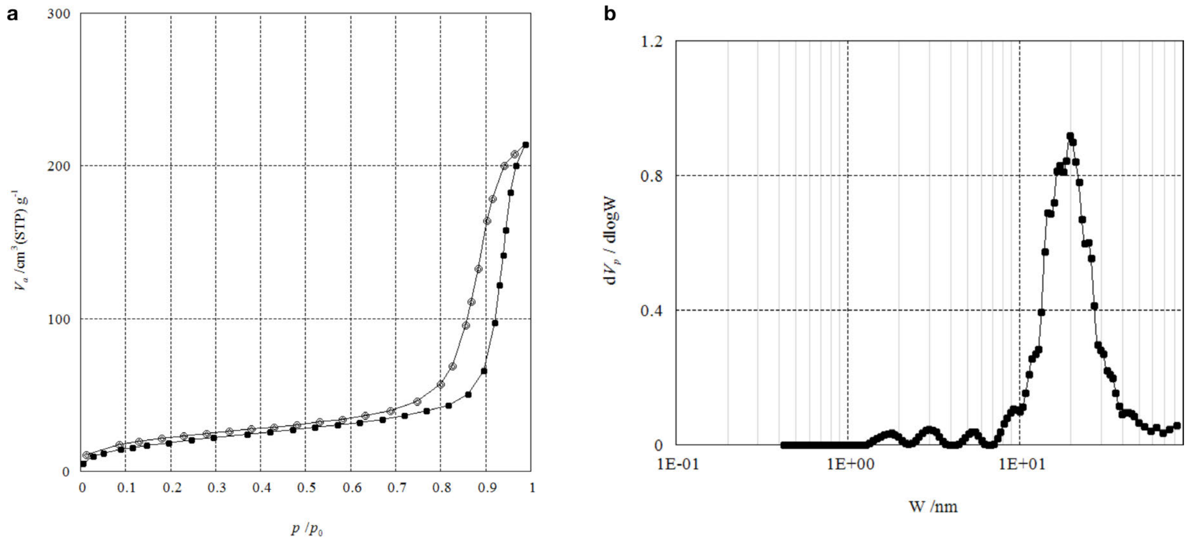

Supplementary Figure 12. (a) N_2 adsorption isotherm of PSt-**1** at 77 K, (closed symbol) adsorption curve; (open symbol) desorption curve. The isotherm shows typical behavior of mesoporous material. (b) Pore-size distribution of PSt-**1** calculated using NLDFT method. W denotes pore width (nm). The distribution has a peak at W = 19.5 nm. The porosimetry results indicate mesoporous nature of PSt-**1** at dried state. The calculated pore size is ~20 nm, which could reflect the film morphology. Since the mesh structure can be collapsed and shrunk at the dried condition, the observed mesoporous nature could be also attributed to the agglomerated morphologies of the polymer particles.

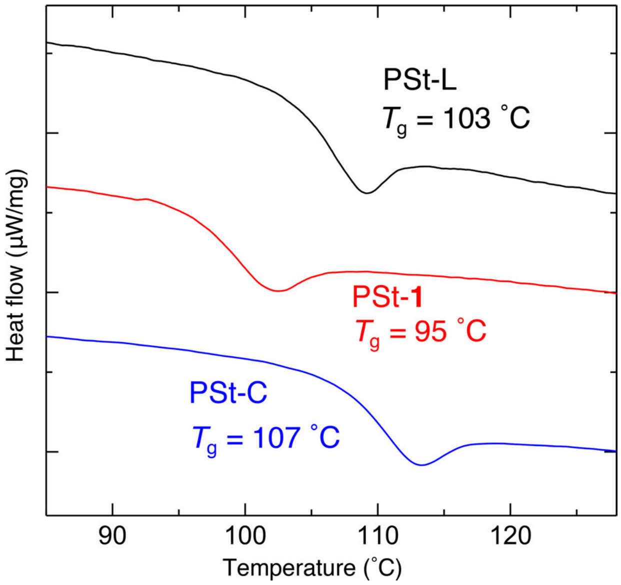

Supplementary Figure 13. DSC curves of PSt-L, PSt-**1**, and PSt-C (heating rate = 10 °C min^{-1}).

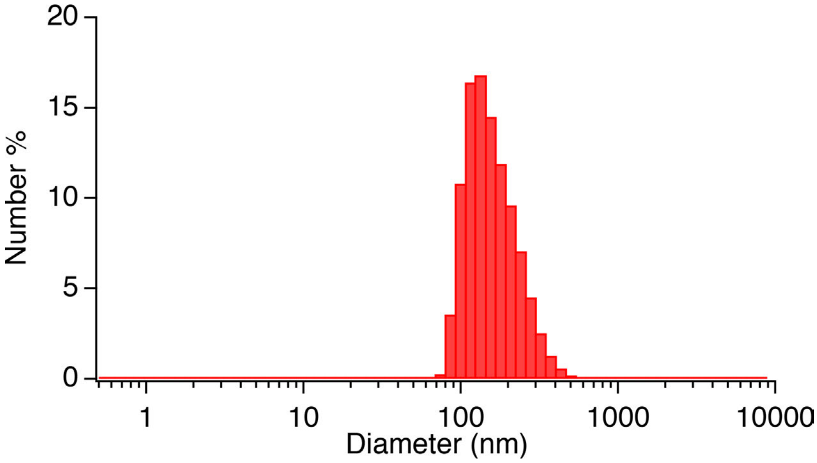

Supplementary Figure 14. Size distribution of PSt-C measured by DLS in chloroform at 25 °C.

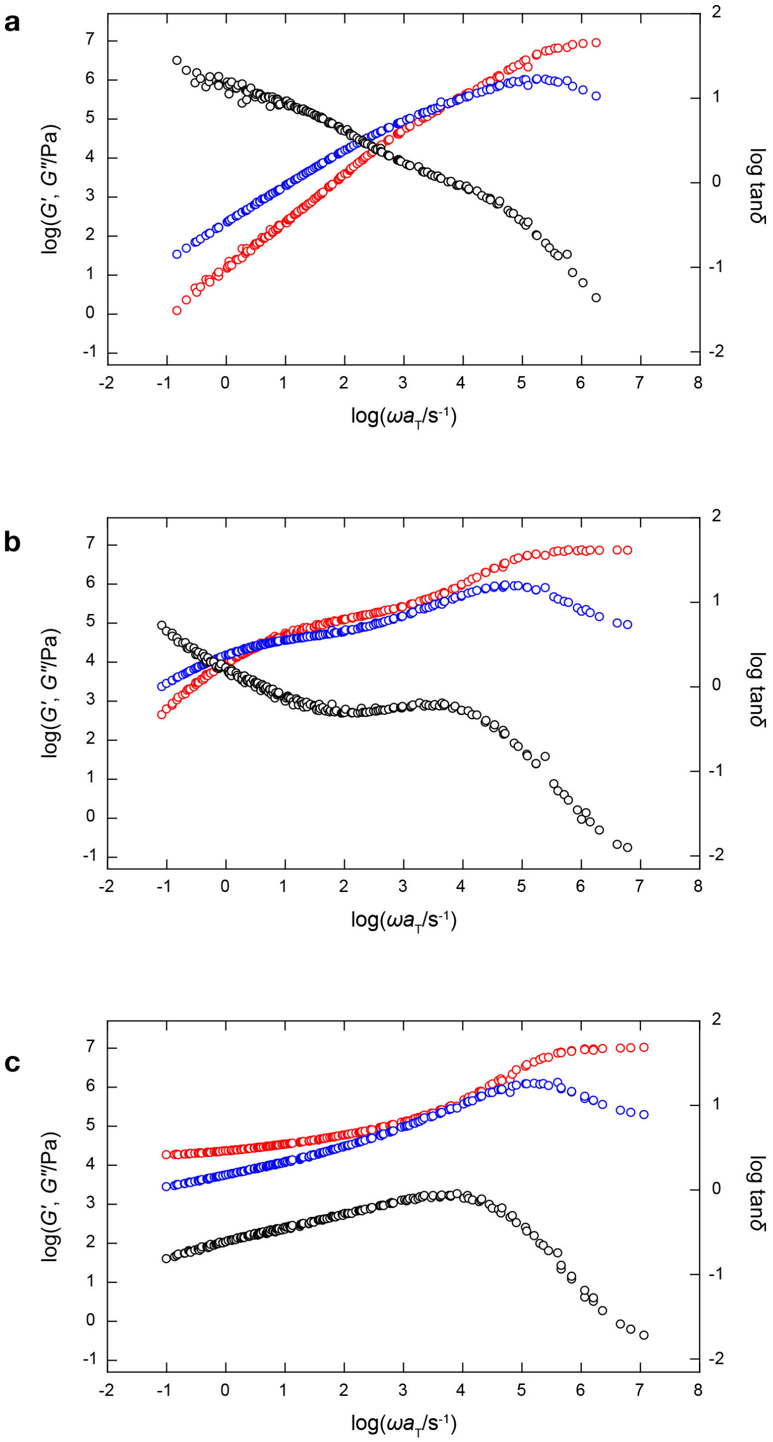

Supplementary Figure 15. Master curves for (a) PSt-**1**, (b) PSt-L, and (c) PSt-C, which are constructed by shifting and superimposing multiple temperature–frequency data with the reference temperature T_{ref} of 160 °C. (red) Storage modulus (G'); (blue) loss modulus (G"); (black) loss factor (tan $\delta = G"/G'$).

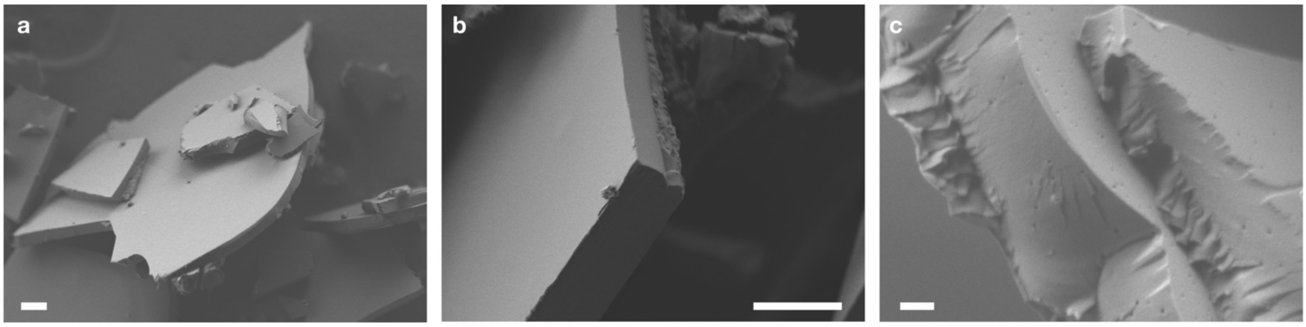

Supplementary Figure 16. SEM micrographs of the PSt-**1** cast film. The film (thickness: ~3 μm) was prepared by casting from its chloroform solution (40 mg mL^{-1}) on a glass substrate. (a) ×700 (Scale bar: 10 μm). (b) ×2,500 (Scale bar: 10 μm). (c) ×9,500 (Scale bar: 1 μm).

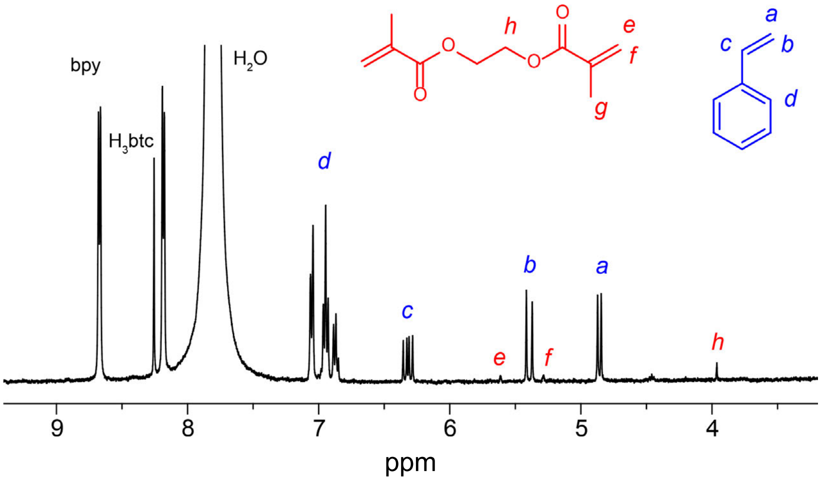

Supplementary Figure 17. ^{1}H NMR spectrum of **1**⊃St digested in DCl/DMSO-d_6 mixture before polymerization reaction. Monomer and EDMA incorporation ratios and conversions were calculated using the integral value of peaks a and h, respectively.

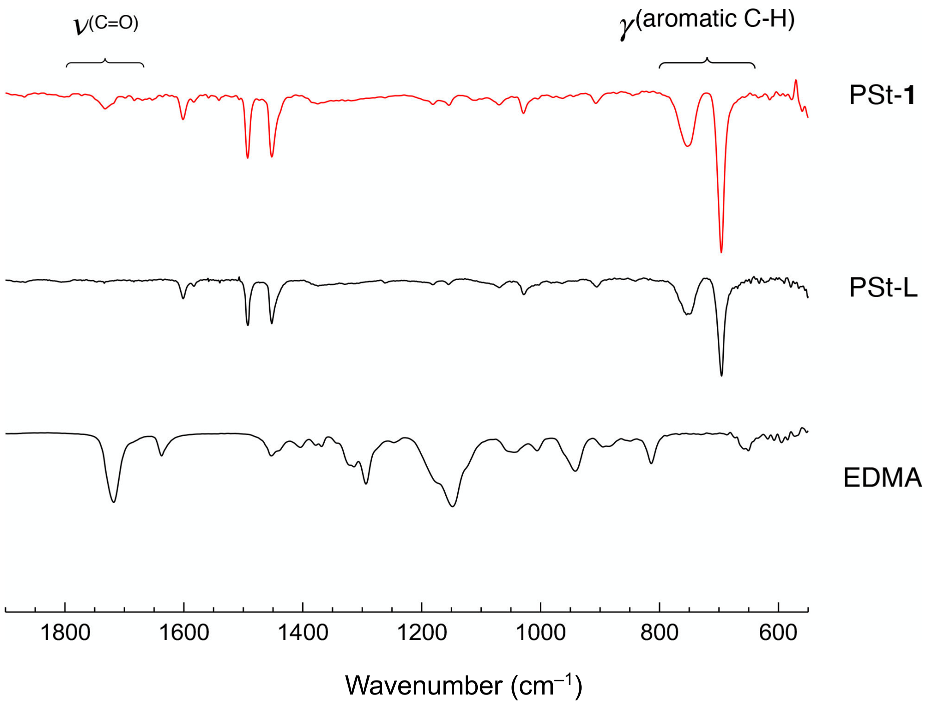

Supplementary Figure 18. FT-IR spectrum of PSt-**1**. FT-IR spectra of PSt-L and EDMA are also given for comparison.

2. Supplementary Methods

General

^{1}H nuclear magnetic resonance (NMR) spectra were recorded using JEOL model ECS-400 spectrometers operating at 400 MHz. SEM measurements were performed using a Hitachi model S-3000N at an accelerating voltage of 5 kV and a JEOL model JSM-7800F Prime at an accelerating voltage of 1.5 kV. Samples were deposited on a conducting carbon tape attached by an SEM grid, then coated with platinum. Atomic force microscopy (AFM) micrographs were obtained in non-contact tapping mode using Asylum Research model Cypher ES. Gold-coated cantilevers (OMCL-AC160TSA-C3E, Olympus) with spring constants ranging from 21 to 28 pN nm^{-1} (resonant frequency of 300 kHz) were used and calibrated by the thermal fluctuation method. Igor Pro software (WaveMetrics) and Gwyddion[1] were used for data acquisition and analysis, respectively. X-ray powder diffraction (XRPD) data were recorded on a Rigaku model SmartLab X-ray diffractometer with Cu Kα radiation. Size-exclusion chromatography measurements were performed at 40 °C on a Shodex model GPC-101 system with two polystyrene gel columns in series (Shodex KF-806M) and equipped with a refractive index detector and an UV detector. The mobile phase was chloroform at a flow rate of 1.0 mL min^{-1}. Molecular weights and molecular weight distribution (M_w/M_n) were obtained relative to polystyrene standards (Shodex, Standard Polystyrene SM–105). Dynamic light scattering (DLS) measurements were performed at 25 °C on Malvern model Zetasizer equipped with a He-Ne laser (λ = 633 nm) using chloroform as the solvent. The light scattering signal was detected at a fixed angle of 173°. Static light scattering (SLS) measurements were performed at 25 °C on ALV model ALV/DLS/SLS-5000 light scattering system at multi angles in the range of 50°-150° using toluene as the solvent. A He–Ne laser (λ = 633 nm) was used as the incident beam. Samples for DLS and SLS measurements were prepared by filtering chloroform solutions through a 0.45 µm PTFE filter into a glass cell with a path length of 1 cm. Differential scanning calorimetry (DSC) was carried out using Hitachi High-Tech Science Corporation model DSC7020 at the heating rate of 10 K min^{-1}. The glass transition temperature was determined by using a software TA7000 Standard Analysis (Ver. 5.0). Energy dispersive X-ray fluorescence (XRF) analysis was performed using Rigaku model NEX-CG at room temperature in a vacuum condition. Fundamental parameter (FP) method was used for the quantitative analysis. N_2 gas adsorption measurements were performed in volumetric method using MicrotracBEL model BELSORP-mini. The sample was dried and evacuated at 80 °C for 16 hours under vacuum prior to the measurements. Non-local density functional theory (NLDFT) simulation for pore distribution analysis was performed using BELMaster software (MictotracBEL), version 6.3.1.0.

Materials

All of the reagents and chemicals used were obtained from commercial sources, unless otherwise noted. 2,2'-Azobis(isobutyronitrile) (AIBN) was recrystallized from methanol solution. Styrene and methyl methacrylate were purified by vacuum distillation prior to use. Ethylene glycol dimethacrylate was

purified by alumina prior to use. Deuterated solvents for NMR spectroscopy were purchased from Cambridge Isotope Laboratories. DCl (35% in D_2O) was purchased from Sigma Aldrich.

Synthesis of PSt-L: Solution-phase free-radical polymerization of St was carried out using AIBN as radical initiator. St (10 mL) and AIBN (60 mg) were placed in 50-mL flask. The mixture was degassed by freeze-pump-thaw cycles (three times) and stirred at 60 °C. After 20 h, the reaction mixture was poured into a large amount of methanol. The white solid was collected and purified by reprecipitation cycles (two times), then dried under reduced pressure to afford PSt-L (4.9 g).

Synthesis of PSt-C: The synthesis of PSt-C was carried out in the similar method reported in the literature.[2] Water solution (90 mL) of surfactant (CTMA: cetyltrimethylammonium bromide) was prepared and degassed by N_2 bubbling for 30 min. Mixture of St (10 g), EDMA (0.19 g), AIBN (50 mg) was prepared separately and degassed in the same manner. Then, both mixtures are mixed and stirred for 1 day at room temperature. The mixture was heated afterward to 60 °C. The reaction was stopped after 3 days. The reaction mixture was poured into a large amount of hot methanol. The crude product thus formed was collected by filtration and redissolved into tetrahydrofuran (THF), then precipitated again in methanol. This precipitation cycle was repeated for three times to carefully remove the surfactant. The powdery product was collected by filtration and dried under reduced pressure at 50 °C, affording PSt-C (8.9 g).

Molecular Dynamics (MD) Simulations

MD simulations were performed using the Materials Studio Modeling v4.4 software package (Accelrys Inc., San Diego, CA, USA) using the Universal Force Field, as implemented in the Forcite module. The charges were dealt with by the charge equilibration method in this system. The initial structure of **1** was generated based on the X-ray crystal structure. The quench dynamics with the optimized structures were conducted at 493 K, and then, MD calculations were carried out at 293 K for 1000 ps under NVT conditions.

Single Crystal X-ray Diffraction Analysis

A single crystal of **2** was mounted using MiTeGen's MicroMount™. Intensity data were collected at 103 K in flowing low temperature nitrogen gas on a Rigaku XtaLAB P200 with VariMax Mo Optic with MoKα radiation (λ = 0.71075 Å) and a confocal monochromator. The structure was solved by direct methods and refined by full-matrix least-squares cycles in SHELX2018/1.[3] All non-hydrogen atoms were refined with anisotropic thermal parameters. Hydrogen atoms attached to C were located at geometrically calculated positions and refined with isotropic thermal parameters. The guest solvent molecules included in the channels showed severe disorder and the SQUEEZE command in PLATON was used in the structure refinement.[4] Crystallographic data for **2** has been deposited with the CCDC (number 1905001). CheckCIF gave 3 level B alerts. All alerts result from disordering problem mostly

originating from the high thermal motion of the central phenyl ring of dpb ligand and free OH group of Hbtc ligand.

Dynamic Mechanical Analysis

Oscillating-shear dynamic mechanical analyses (DMA) for polystyrene samples were carried out using UBM Japan model Rheosol-G5000 with a parallel plate (ϕ = 20 mm). Measurement temperature was varied in the range of 200-50 °C. Frequency range of 0.1-20 s^{-1} was applied in a linear strain region of 1% at each temperature point. Samples for DMA were annealed at 120 °C for 16 h prior to the measurement. After mounting in the DMA apparatus, the samples were further annealed at 200 °C for 5 min to have full relaxation of materials. Master curves of the dynamic storage (G') and loss (G'') moduli were constructed by the time–temperature superimposition with the reference temperature of T_{ref} = 160 °C. Specifically, the frequency data at each temperature were horizontally shifted by a_T to have the best fitting curves.

3. Supplementary References

1. Nečas, D. & Klapetek, P. Gwyddion: an open-source software for SPM data analysis. *Cent. Eur. J. Phys.* **10**, 181–188 (2012).
2. Antonietti, M. *et al.* Synthesis and size control of polystyrene latices via polymerization in microemulsion. *Macromolecules* **24**, 6636–6643 (1991).
3. Sheldrick, G. A short history of SHELX. *Acta Crystallogr.* **A64**, 112–122 (2008).
4. A. L. Spek, Single-crystal structure validation with the program PLATON. *J. Appl. Cryst.* **36**, 7–13 (2003)
5. Orts, W. J., Van Zanten, J. H., Wu, W. L. & Satija, S. K. Observation of temperature dependent thicknesses in ultrathin polystyrene films on silicon. *Phys. Rev. Lett.* **71**, 867–870 (1993).
