# Supplementary material for: Unimolecularly thick monosheets of vinyl polymers fabricated in metal–organic frameworks
Source: Nat Commun. 2020 Jul 17;11:3573. doi: 10.1038/s41467-020-17392-1 (PMC7367882; doi:10.1038/s41467-020-17392-1)
Supplement: Supplementary file 3 — Description of Additional Supplementary Files [file 41467_2020_17392_MOESM3_ESM.pdf]

Description of Additional Supplementary Files

Supplementary Movie 1. The visual appearance of PSt-**1** and PSt-L at 150 °C. PSt-**1** (right) is much softer compared to its linear analogue, PSt-L (left). PSt-**1** shows liquid-like behavior while PSt-L appears like an elastic solid.
